# Treatment of posterior Benign Paroxysmal Positional Vertigo is efficient and safe with a new Mechanical Rotational Chair

**DOI:** 10.3389/fneur.2023.1239959

**Published:** 2023-08-17

**Authors:** Dan Dupont Hougaard, Kirsten Duch, Mathias Winther Bech

**Affiliations:** ^1^Balance & Dizziness Centre, Department of Otolaryngology, Head & Neck Surgery and Audiology, Aalborg University Hospital, Aalborg, Denmark; ^2^Department of Clinical Medicine, Aalborg University, Aalborg, Denmark; ^3^Unit of Clinical Biostatistics, Aalborg University Hospital, Aalborg, Denmark

**Keywords:** Benign Paroxysmal Positional Vertigo (BPPV), mechanical rotational chair, Rotundum, vertigo, repositioning chair

## Abstract

**Background:**

Benign paroxysmal positional vertigo (BPPV) is the most predominant vestibular disease. Previously, in the management of BPPV, both therapeutic and diagnostic benefits with mechanical rotation chairs (MRCs) have been reported. However, no previous studies have examined the efficacy of MRC treatment with a fairly new MRC.

**Methods:**

Randomized three-armed parallel open-labeled prospective clinical trial. One hundred and five patients diagnosed with posterior BPPV underwent diagnostics and treatment with an MRC. Patients were randomized to either a Semont maneuver, an Epley maneuver or a 360-degree backwards somersault maneuver. Primary endpoint was to evaluate the efficiency of an MRC in the treatment of posterior BPPV with three separate treatment modalities. Secondary objectives included subjective vertigo assessments.

**Results:**

BPPV treatment with this MRC is very efficient with success rates up to 97.1 percent. The number of treatments required to achieve complete resolution of both objective findings and subjective symptoms was 1.5. Almost 47 percent of patients experienced complete resolution of both subjective and objective measures following one (first) treatment. All Dizziness Handicap Inventory scores decreased significantly post-treatment.

**Conclusion:**

Treatment of posterior BPPV, with the MRC used in this study, was very efficient with both the Semont, the Epley, and the 360-degree backwards somersault maneuver. Based on the findings in this study, this fairly new MRC seems both effective and safe to use.

## Introduction

With a cumulative lifetime incidence close to 10% at the age of 80, benign paroxysmal positional vertigo (BPPV) is the most common cause of vertigo (1). The disease is characterized by abrupt, fleeting spells of vertigo with characteristic nystagmus patterns that happen in response to head position changes with respect to gravity (2). Symptoms can range from sporadic incidences of moderate vertigo without concomitant vegetative symptoms to severe episodes of debilitating vertigo with accompanying nausea and vomiting that considerably impair daily life activities (3).

Benign Paroxysmal Positional Vertigo is believed to be caused by the dislodgement of otoconia from the utricular macula into one or several of the semicircular canals (SCCs) within the inner ear. The dislodged otoconia are moved by gravitational forces when these forces act in the same plane as the affected SCC, disturbing endolymphatic flow (4). Endolymphatic debris is thought to affect cupular dynamics in two different ways: either by floating freely within the endolymphatic space of the SCC(s), a condition known as canalolithiasis (CAN), or by adhering to the cupula of the SCC(s), known as cupulolithiasis (CUP) (5).

BPPV is diagnosed by positional tests in which the patient is placed in specific head positions meant to align the planes of the examined SCCs with the force of gravity. In patients with BPPV, this elicits both an objective recognizable positional nystagmus and a concurrent subjective sensation of vertigo (6). Similarly, BPPV is treated by placing the patients in sequences of head positions that relocate the displaced otoliths back into the utricle. These maneuvers, termed canalith reposition procedures (CRPs), are most frequently performed on an examination bed. These CRPs are highly effective, as success rates have been reported to range between 80 and 90 percent (7). The efficacy of CRPs seems to vary significantly depending on the BPPV subtype and -localization. Especially the CUP subtype BPPV together with horizontal- and multi-canal BPPV have shown greater resistance to traditional CRPs than the most common type of BPPV, posterior CAN (8, 9).

Despite the relatively high success rates reported for traditional CRPs, 10–20 percent of patients undergoing BPPV treatment remain afflicted by the condition following several treatment attempts. Others are unable to undergo traditional CRPs in the first place, i.e., due to cervical spine issues (10). Since approximately 86 percent of patients with BPPV face lost days at work and interruptions of daily life activities due to BPPV, intractable cases constitute a significant healthcare burden in addition to the individual distress caused by the condition (11).

In recognition of these challenges, several mechanical repositioning chairs (MRCs) have been developed. These MRCs allow patients to be rotated 360 degrees in two or three planes while they remain fixed in the sitting position. Mechanical repositioning chairs facilitate complete examiner control of the angles of head positioning, as well as the speed of individual head movements, thus enabling precise and replicable diagnostic procedures in patients who would otherwise be unable to cooperate. Mechanical repositioning chairs also enable specialized replicable therapeutic maneuvers that can only be performed with an MRC (2, 10).

Three types of MRCs are currently being used across Europe: The Epley Omniax Rotator^®^ (Vesticon^©^, Portland, United States) (E-MRC), the TRV^®^ Chair (Interacoustics^©^, Middelfart, Denmark) (T-MRC), and, more recently, the Rotundum^®^ rotary chair (balcare GmbH, Küsnacht, Switzerland) (R-MRC). The mentioned MRCs are all supplemented with VideoNystagmoGraphy (VNG) equipment, allowing precise monitoring of eye movements with added features such as automatic pupil tracking, nystagmus slow-phase velocity measurements, and video recordings for reevaluations (11). The MRC used in this present study allows exact positioning (one-degree intervals) in the yaw- and roll axes and has the benefit of being portable. This last feature enables setting up an advanced mobile clinic for BPPV diagnostics and treatment. In a previous study, this feature was utilized by taking a mobile clinic containing this MRC to several retirement homes. Just above 10% of residents, who experienced dizziness presently or in the recent past, were diagnosed with BPPV. All patients were successfully treated with this MRC. This highlights the potential benefits of implementing close-to-the-citizen BPPV diagnostics and treatment by means of a portable MRC set-up that allows the health workers to go to the patient instead of having the patient go to the out-patient clinic (12).

Though previously published studies have indicated both diagnostic and therapeutic benefits to the use of MRCs in the diagnostics and treatment of BPPV, at this point in time, the amount of research on the matter remains somewhat modest (13–15). To the knowledge of the authors, the fairly new MRC used in this study, has as only been utilized in one single published peer-reviewed study (12).

The primary objective of this study was to evaluate the efficiency of a fairly new MRC in the treatment of posterior BPPV with patients referred directly from General Practitioners (GPs). Secondary objectives included comparisons of the efficiency of the three individual maneuvers used with this MRC. Following a diagnosis of posterior BPPV, treatment options with this MRC included: (1) the Epley maneuver, (2) the Semont maneuver, and (3) the 360-degree backwards somersault maneuver (16). Treatment efficiency was evaluated according to (1) the number of treatments required to achieve complete resolution of both objective findings as well as subjective symptoms, (2) the amount of subjective relief in total and with three separate treatment modalities, and (3) complete resolution following one (first) treatment. Tertiary objectives included subjective vertigo assessment with fulfillment of the 25-item Dizziness Handicap Inventory (DHI) questionnaire for individuals who were treated successfully. The assessment included comparisons of (1) pre- and post-treatment total and subcategory scores and (2) differences between pre- and post-treatment total and subcategory scores. Quaternary objectives included evaluation of treatment failures and registration of any adverse- or serious adverse events.

## Materials and methods

### Participants

The study population consisted of adult patients referred directly from GPs to the tertiary Balance and Dizziness Centre at the Department of Otolaryngology, Head and Neck Surgery and Audiology at Aalborg University Hospital, Denmark. All patients referred had a classical BPPV case history and/or a clinical presumption of BPPV at the time of referral. None of the referred patients had received any BPPV treatment prior to referral.

### Methods

All participants included in the study were diagnosed with posterior BPPV following a positive Dix-Hallpike (DH) test where up-beating and torsional positional nystagmus was observed along with a concomitant subjective spinning/vertiginous sensation. Bilateral Supine Roll Tests (SRTs) were also performed to rule out BPPV in any of the lateral SCCs. Following inclusion and one treatment at the initial visit, all participants were scheduled for one in-house follow-up 2 to 4 weeks after their first visit. At this follow-up visit, participants underwent diagnostic positional testing with the DH test as well as the SRT. If no BPPV was diagnosed, no further treatments were given. If positional nystagmus, pathognomonic for posterior BPPV of the previously affected SCC, was observed in the ipsilateral DH position, continuous treatment was offered with every follow-up visit throughout the study period with 2-to-4 week intervals consistently between treatments. In case concomitant lateral BPPV was diagnosed, posterior BPPV was treated first. Following successful treatment of the posterior BPPV, additional treatment(s) were offered to relieve the participant of his/her lateral BPPV (not included in the protocol). Following complete remission of objective findings and subjective symptoms compatible with BPPV, a 25-item DHI-questionnaire was filled out. This questionnaire was also filled out at the time of inclusion.

At their final follow-up visits, every participant was asked whether their BPPV-related vertiginous symptoms had been cured, alleviated, unaffected or worsened following treatment in the R-MRC. Subjective relief of symptoms was defined by patients stating that their episodic vertiginous symptoms had been alleviated of cured following targeted treatment in an R-MRC.

Prior to inclusion, all patients underwent screening tests for spontaneous- and gaze-evoked nystagmus as well as a rotation test including VOR suppression. Patients with abnormal findings on these screening tests were excluded from the study. Since all patients included presented with a typical BPPV case history and had to display nystagmus patterns compatible with BPPV to receive the BPPV diagnosis, extensive neurological examinations were not routinely performed.

### Materials

Diagnostic tests in the MRC were performed with the aid of VNG goggles. The diagnostic tests were meticulously conducted in the following order for every patient regardless of findings: left DH test, right DH test, supine position, left SRT, and right SRT. Individual positions with both the DH tests and the SRTs were maintained for 60 s. All participants were diagnosed and treated with the Rotundum^®^ rotatory chair (balcare^©^, Küsnacht, Switzerland) (R-MRC). Participants were fitted with VertiGoggles^®^ (balcare^©^, Küsnacht, Switzerland). The accompanying software (VertiPACS^®^, GDT version (3.1), balcare^©^, Küsnacht, Switzerland) enabled quantification and characterization of nystagmus with analysis of parameters such as direction (horizontal, vertical, and/or rotational) as well as average slow phase velocities. Patients diagnosed with BPPV were classified as either primary or secondary BPPV based upon their etiology. Secondary BPPV included patients with a recent head trauma (time wise correlation with onset of vertiginous symptoms) or patients with concurrent inner ear disease(s) that predispose(s) to the development of BPPV. Patients with no clear etiology were classified as primary or idiopathic BPPV.

### In- and exclusion criteria

Inclusion criteria included: (1) age at or above 18, (2) classic BPPV case history (a spinning illusory sensation (vertigo) produced by changes in head position relative to gravity), (3) objective findings with observation of posterior BPPV characteristic positional nystagmus (combined up-beating and rotational positional nystagmus) with the Dix-Hallpike test, (4) minimum requirement of unilateral posterior SCC affection (both CAN and CUP BPPV subtypes were included). Exclusion criteria included: (1) age below 18, (2) treatment of BPPV with an MRC within the last 6 months, (3) treatment of BPPV by a health care professional within the last month, (4) withdrawal of the BPPV diagnosis, (5) off-protocol treatment, (6) known cerebral aneurism or previous cerebral hemorrhage, (7) insufficient cooperation during diagnostics and/or treatment, and (8) treatment with sedative antihistamine within the last 7 days of examination.

### Study design

**Figure 1:**
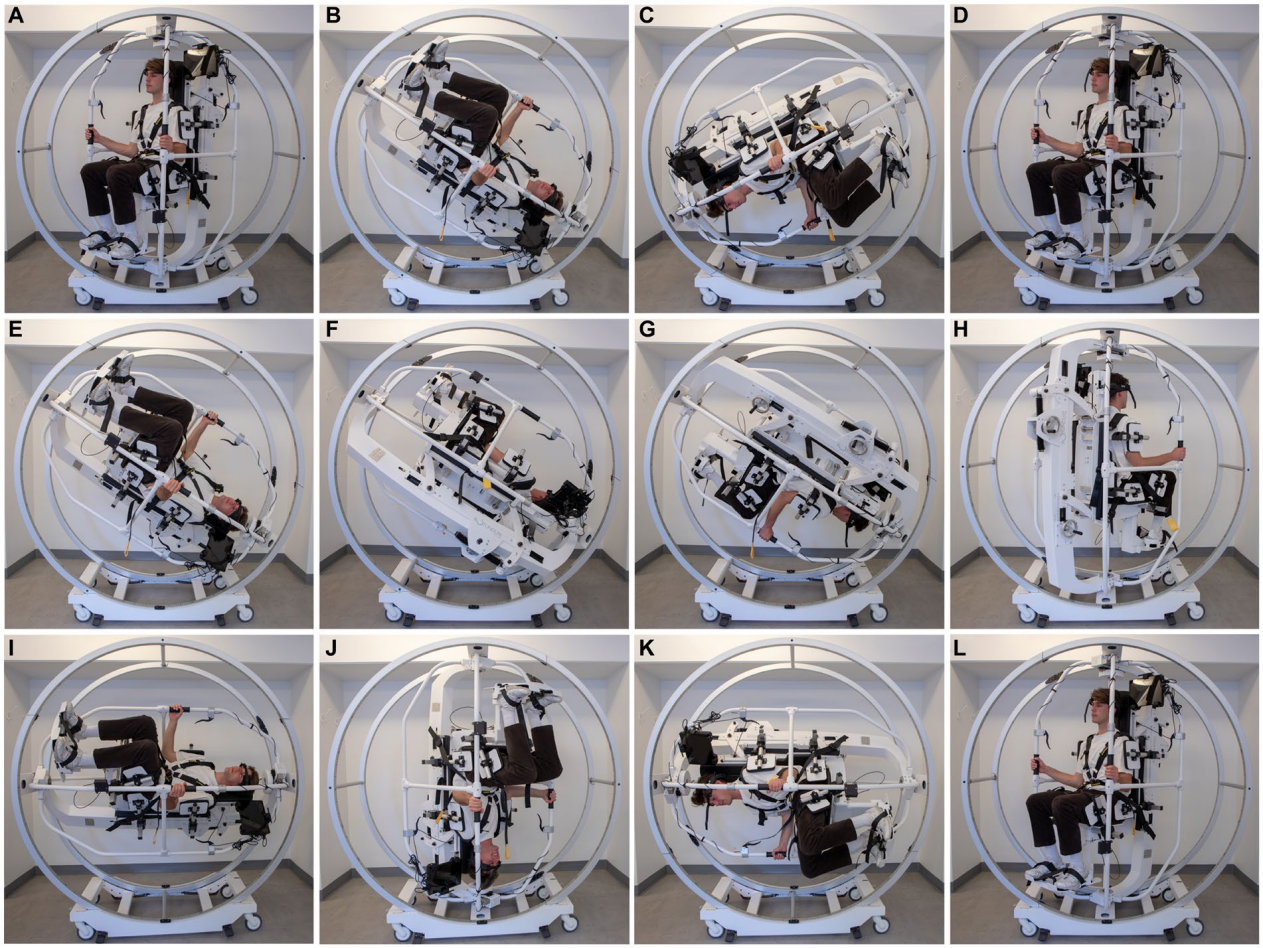
Semont maneuver **(A**–**D)**: this CRP is initiated with the patient upright and turned 45 degrees in the yaw axis towards the affected side **(A)**. Then the patient is moved 120 degrees backwards in the roll axis on the affected side **(B)**. This position is kept for 30 s. Then the MRC is rotated fast in the roll axis a total of 240 degrees towards the healthy side **(C)**. This position is also kept for 30 s. The CRP is completed when the patient is positioned upright from this position **(D)**. Epley maneuver **(E**–**H)**: this CRP is initiated with the patient upright and turned 45 degrees in the yaw axis towards the affected side **(A)**. Then the patient is moved 120 degrees backwards in the roll axis **(E)**. This position is kept for 30 s. Then the patient is turned 90 degrees towards the healthy side in the yaw axis **(F)**. This position is kept for 30 s. An additional 90 degree turn towards the healthy side follows and this position is again held for 30 s **(G)**. The CRP is completed when the patient is positioned upright from this position **(H)**. The 360-degree somersault maneuver **(I**–**L)**: this CRP is initiated with the patient upright and turned 45 degrees in the yaw axis towards the affected side **(A)**. Then the patient is moved 90 degrees backwards in the roll axis on the affected side. This position is kept for 30 s **(I)**. Then the patient is then continuously moved backwards in the pitch axis for an additional 270 degrees **(J**–**L)**. The CRP is completed when the patient is positioned upright in the starting position and has been rotated a total of 360 degrees backwards **(L)**. The total duration of the 360-degree rotation was approximately 1 minute. Please note that all illustrations show treatments targeted left-sided posterior BPPV and please also note that the initial position with the Epley maneuver and the 360-degree somersault maneuver is not shown but is the same as the starting position with the Semont maneuver **(A)**. Please also note that an upright starting position with the patient facing straight left is a prerequisite for correct repositioning if following the above mentioned instructions.

The study was conducted as a Prospective Randomized Clinical Trial (RCT). Benign paroxysmal positional vertigo was diagnosed and subcategorized according to the Bárány criteria (6). Following inclusion, all patients underwent 1:1:1 randomization for continuous treatment with one of three offered treatments: (1) the Epley maneuver, (2) the Semont maneuver, or (3) the 360-degree somersault maneuver. Please refer to Figure 1 for details on the three maneuvers. Consecutive treatments with the designated maneuver (chosen by randomization), were continued until complete remission of objective findings was observed and subjective vertiginous symptoms was reported by the participant during positional testing or until a maximum of ten identical treatments proved unsuccessful.

### Ethics, data management, and statistics

The study was approved by the North Denmark Region Committee on Health Research Ethics, ID no. N-20190054. Patient characteristics at baseline were presented according to sample distribution with means and standard deviations (SD), medians and interquartile ranges (IQR) or counts and percentages. The mean number of treatments required for successful treatment was presented and compared for patients experiencing relief or remission. The percentage of successful treatments across treatments was presented and compared after the first treatment and following the entire treatment plan. Finally, the pre- and post-treatment DHI-scores were presented for patients who were treated successfully and changes in these scores were compared across groups. Complete data analysis was used in the few analyses where data was missing. All statistical work was done by a certified biostatistician.

## Results

A total of 508 patients were referred directly from participating general practitioners with a case history compatible with BPPV. Four hundred and three of patients referred were not included in the study, the vast majority (83%) because diagnostic criteria for BPPV were not met. Other reasons included lack of cooperation for diagnostics/treatments with the R-MRC, withdrawal of study consent, or a diagnosis of exclusively non-posterior BPPV. Therefore, a total of 105 participants were included in the study. Thirty-seven of these were randomized to undergo treatment with the Semont maneuver, 34 were randomized to undergo treatment with the Epley maneuver, and 34 were allocated to the group of patients undergoing treatment with the 360-degree somersault maneuver. For details on the background characteristics, please refer to Table 1.

**Table 1:** Baseline characteristics.

	Semont (*n* = 37)	Epley (*n* = 34)	360 (*n* = 34)	Total (*n* = 105)	*p*-Value
Age, years					
Mean (SD)	60.3 (15.5)	61.2 (14.2)	55.1 (18.8)	58.9 (16.3)	0.25
**Gender**, number (%)					
Female	26 (36.1)	22 (30.6)	24 (33.3)	72 (100.0)	0.90
Male	11 (33.3)	12 (36.4)	10 (30.3)	33 (100.0)	
**Previous BPPV**, number (%)					
Yes	7 (24.1)	12 (41.4)	10 (34.5)	29 (100.0)	0.33
No	30 (39.5)	22 (28.9)	24 (31.6)	76 (100.0)	
**Duration**, days (SD)					
Mean	46.6 (98.5)	117.6 (258.1)	96.2 (305.8)	85.6 (234.7)	0.42
Median [iqr]	18.0 [9,31]	24.5 [12.5,64.5]	21.0 [9.0,63.5]	21.0 [9,63]	
Range	3–586	2–1411	2–1799	2–1799	
**Etiology**, number, (%)					
Primary	37 (37.4)	33 (33.3)	29 (29.3)	99 (100.0)	0.02
Secondary	0 (0.00)	1 (16.7)	5 (83.3)	6 (100.0)	
**BPPV subtype**, number (%)					
Canalolithiasis	33 (33.3)	34 (34.3)	32 (32.3)	99 (100.0)	0.13
Left	10 (27.8)	14 (38.9)	12 (33.3)	36 (100.0)	
Right	23 (36.5)	20 (31.7)	20 (31.7)	63 (100.0)	
Cupulolithiasis	4 (80.0)	0 (0.00)	1 (20.0)	5 (100.0)	
Left	2 (100.0)	0 (0.00)	0 (0.00)	2 (100.0)	
Right	2 (66.67)	0 (0.00)	1 (33.3)	3 (100.0)	
**Total DHI-score**, mean (SD)					
Pre-treatment	40.6 (17.7)	38.7 (16.3)	42 (18.3)	40.4 (17.3)	0.74

Selected population characteristics are provided with all three BPPV subgroup by numbers, means and medians. Percentages, where provided, compare treatment subgroup numbers with the entire study population for easier comparison. Please note that all baseline characteristics did not differ significantly between subgroups except etiology. Because age and duration of BPPV related symptoms were not normally distributed, medians are also provided with these characteristics. Duration is defined as the time from the onset of BPPV related symptoms until the first visit. The subgroup with secondary BPPV includes traumatic BPPV, vestibular neuritis, Meniere’s disease, and patients with previous ear surgery. Please note that BPPV subtype data is missing from one person in the 360-degree subgroup. Significant value of *p*s are provided in bold. 360, 360-degree somersault maneuver subgroup.

The total number of required treatments was 1.5 in average. The number of required treatments did not differ significantly between individual treatment subgroups. When evaluating complete remission of positional nystagmus (objective remission) and complete remission of subjective symptoms (subjective remission), there were no significant differences between treatment subgroups in terms of the number of required treatments before successful treatment was achieved. Please refer to Table 2.

**Table 2:** Numbers of treatments required before successful treatment.

	Semont (*n* = 37)	Epley (*n* = 34)	360 (*n* = 34)	Total (*n* = 105)	*p*-Value
Average number of treatments given, mean (SD)	1.3 (0.6)	1.6 (1.3)	1.7 (1.4)	1.5 (1.2)	0.38
Objective success (complete remission of positional nystagmus), mean (SD), *n* = 88	1.4 (0.7)	1.5 (1.4)	1.6 (0.9)	1.5 (1.0)	0.71
Subjective success (complete remission of subjective symptoms), mean (SD), *n* = 71	1.3 (0.6)	1.3 (0.7)	1.6 (0.9)	1.4 (0.7)	0.30

Most patients experienced concomitant remission of both objective findings and subjective symptoms. The number of required treatments did not differ significantly when defining successful treatment exclusively by complete remission of either objective findings or subjective symptoms.

Overall, 66 out of 105 (62.9%) experienced complete resolution of both objective findings and subjective BPPV-related symptoms. Following one treatment, the Epley maneuver performed significantly better than the two other treatments being examined in this study. However, there were no significant differences in successful treatment rates between the three treatment subgroups over time and none of the patients included required more than four treatments in total. Please refer to Table 3.

**Table 3:** Complete resolution of BPPV-related objective findings and subjective symptoms.

Number of treatments	Semont (*n* = 37)	Epley (*n* = 34)	360 (*n* = 34)	Total (*n* = 105)	*p*-Value
One	18 (48.6)	21 (61.7)	10 (29.4)	49 (46.7)	**0.03**
Two	3 (8.1)	2 (5.9)	4 (11.8)	9 (8.6)	
Three	2 (5.4)	2 (5.9)	3 (8.8)	7 (6.7)	
Four			1 (2.9)	1 (1.0)	
Overall	23/37 (62.2)	25/34 (73.5)	18/34 (52.9)	66/105 (62.9)	0.21

The number of patients with complete remission of both objective findings AND subjective symptoms are listed as well as percentages (parenthesis). Please note that the Epley maneuver is the most efficient treatment modality after one treatment (significant) but also note that overall success rates did not differ significantly between subgroups. The 360-degree somersault maneuver seems to be the least efficient repositional maneuver, especially after one treatment. That tendency is, however, non-significant. Significant value of *p*s are provided in bold. 360, 360-degree somersault maneuver.

Overall, 99 out of 105 (94.3%) experienced either relief or complete remission of BPPV related symptoms following treatment. Overall, there were no significant differences between subgroups. Please refer to Table 4 for further details.

**Table 4:** Relief or complete remission of subjective BPPV-related symptoms.

Number of treatments	Semont (*n* = 37)	Epley (*n* = 34)	360 (*n* = 34)	Total (*n* = 105)	*p*-Value
One	26 (70.3)	24 (70.6)	20 (58.8)	70 (66.7)	0.50
Two	5 (13.5)	4 (11.8)	7 (20.6)	16 (15.2)	
Three	3 (8.1)	4 (11.8)	3 (8.8)	10 (9.5)	
Four			1 (2.9)	1 (1.0)	
Eight		1 (2.9)	1 (2.9)	2 (1.9)	
Overall	34/37 (91.9)	33/34 (97.1)	32/34 (94.1)	99/105 (94.3)	0.64

The number of patients with either relief or complete remission of subjective symptoms are listed as well as percentages (parenthesis). Please note that 99 out of 105 (94.3%) overall experienced relief of subjective symptoms independent of the assigned treatment maneuver and that there were no significant differences between the reported subjective relief/remission rates between the individual treatment subgroups. Also note that two patients required a total of eight treatments (one in the Epley- and one in the 360-degree subgroup). 360, 360-degree somersault maneuver.

Exclusively patients with complete remission of both objective findings and subjective symptoms were included in the tables with results from the DHI questionnaire pre- and posttreatment. All subgroup- and total DHI-scores decreased significantly with all three treatment subgroups following treatment. Please refer to Table 5 for further details.

**Table 5:** Pre- and posttreatment dizziness handicap inventory scores.

	Pre-treatment Semont (*n* = 23)	Post-treatment Semont (*n* = 22/23)	Pre-treatment Epley (*n* = 25)	Post-treatment Epley (*n* = 24/25)	Pre-treatment 360 (*n* = 18)	Post-treatment 360 (*n* = 17/18)	Pre-treatment Total (*n* = 66)	Post-treatment Total (*n* = 63/66)
Physical	15.8 (4.7)	1.0 (2.0)	15.8 (3.8)	1.9 (2.8)	17.7 (5.0)	2.4 (5.3)	16.3 (4.5)	1.7 (3.5)
Emotional	8.6 (5.7)	0.8 (2.4)	8.6 (6.8)	1.2 (2.5)	10.8 (8.2)	0.9 (2.2)	9.2 (6.8)	1.0 (2.4)
Functional	11 (6.2)	0.8 (2.4)	15.2 (8.4)	1.8 (3.2)	15.3 (8)	1.5 (3.6)	13.8 (7.8)	1.4 (3.0)
Total	35.4 (12.6)	2.6 (6.2)	39.6 (16.7)	4.9 (7.1)	43.8 (19.3)	4.8 (10.8)	39.3 (16.3)	4.1 (7.9)

Please note that all post-treatment total DHI-scores fall within the category of “no dizziness handicap” (total DHI-score below 16) but no subgroup- or total DHI-scores drop to zero. Even though not provided in the table, please also note that no pre- or posttreatment means differ significantly between the three treatment subgroups. Mean DHI-scores are provided together with standard deviations (in parenthesis).

All subcategory and total DHI-scores were significantly lower following successful treatment. Additionally, no significant differences were seen between the three treatment subgroups in terms of both subcategory- and total DHI-scores. Please refer to Table 6 for further details.

**Table 6:** Pre- and posttreatment dizziness handicap inventory score differences.

Patients with complete remission	Semont (*n* = 22/23)	Epley (*n* = 24/25)	360 (*n* = 17/18)	Total (*n* = 63/66)	*p*-Value
Physical	15.2 (13.4;17.0)	13.8 (12.1;15.6)	15.5 (11.8;19.3)	14.8 (13.4;16.1)	0.56
Emotional	7.5 (4.9;10.2)	7.2 (4.7;9.6)	10.2 (6.3;14.1)	8.1 (6.4;9.8)	0.32
Functional	10.1 (7.3;12.8)	13.3 (10.2;16.5)	14.1 (10.1;18.1)	12.4 (10.5;14.3)	0.20
Total	32.8 (27.0;38.6)	34.3 (28.2;40.5)	39.9 (29.6;50.2)	35.3 (31.2;39.5)	0.40

Please note that there are no significant differences between the three treatment subgroups (neither total DHI-scores nor subgroup DHI-scores). Mean DHI-scores and standard deviation (in parenthesis) are provided. Please also note that DHI questionnaire fulfilment was missing/incomplete in 3 out of 66 patients (4.5%) with complete objective and subjective remission.

Overall, six patients (5.7%) experienced no subjective relief or remission of BPPV-related symptoms, eight patients (7.6%) had no relief or remission of objective findings, and two patients (1.9%) had neither subjective nor objective relief or remission following ten treatments. Numbers were too low to determine if subgroup differences were significant. Please refer to Table 7 for further details. There were no adverse or serious adverse events in relation to this study.

**Table 7:** Treatment failures.

	Semont (*n* = 37)	Epley (*n* = 34)	360 (*n* = 34)	Total (*n* = 105)
No remission or relief of subjective symptoms (persisting positional vertigo)	3 (2.9)	1 (1.0)	2 (1.9)	6 (5.7)
No remission or relief of objective findings (persisting positional nystagmus)	1 (1.0)	3 (2.9)	4 (3.8)	8 (7.6)
Neither subjective nor objective remission/relief (persisting positional vertigo and -nystagmus)	0 (0.0)	1 (1.0)	1 (1.0)	2 (1.9)

Treatment failures were defined as patients who were not successfully treated after a total of ten treatments. Please note that numbers are low, especially considering the patients that neither had subjective nor objective relief: 2 out of 105 patients (1.9%). Also note that, even though not included in this table, 4 out of 6 (66.7%) patients in the group with no remission or relief of subjective BPPV-related symptoms and 6 out of 8 (75%) patients in the group with no remission or relief of objective findings had canalolithiasis subtype BPPV.

## Discussion

This study showed that the R-MRC was able to treat posterior BPPV efficiently. Depending on the criteria defining successful treatment, a mean of 1.4 to 1.5 treatments were required. All three treatments provided very high success rates with 90+ percentages of participants experiencing relief of their subjective BPPV-related vertiginous symptoms following treatment(s).

Numerous studies have been carried out with the purpose of determining diagnostic and therapeutic properties related to BPPV management. Traditional BPPV diagnostics and treatments are therefore quite thoroughly described, well understood, well researched, and as a direct result hereof, widely accepted. Since the natural history of the BPPV includes very high spontaneous remission rates as well as high numbers of relapses following successful treatment(s) (3, 5, 8), it is the opinion of the authors of this article, that for interstudy comparisons, it is of paramount importance, that BPPV populations *across* studies are similar. The BPPV subtype of CUP as well as bilateral and/or multicanal BPPV have proven more difficult to diagnose and more resilient to treatment (2, 7). Even though these different BPPV disease properties may alter or skew the results significantly, numerous studies insufficiently report their BPPV population characteristics in terms of detail and in uniformity. Additionally, use of different diagnostic criteria (6, 11), combined with inconsistent reporting of the diagnostic criteria actually being used with individual studies, further compromise or limit interstudy comparisons. The fact that the number of treatments given per treatment session varies between one and four (13, 17) further adds to the heterogeneity of MRC study designs. Last, but not least, no consensus exists on a clear definition of successful treatment. Successful treatment may be defined as complete or partial remission of objective findings alone, complete or partial remission of subjective symptoms alone, or a combination of both. Moreover, some BPPV studies even fail to report their criteria for successful treatment. All of the abovementioned limitations and considerations must be taken into account when making BPPV interstudy comparisons.

The use of MRCs in the diagnostics and treatment of BPPV is increasing worldwide. Even though this type of equipment is becoming more and more widespread, evidence related to the diagnostic and treatment related properties remains limited. Up until now, most peer-reviewed studies on MRCs have looked at *treatment efficiency* related to the use of the T-MRC (16) and to a minor extent also the diagnostics properties of this MRC (7). Publications on the R-MRC are almost non-existing. Overall, this study was designed to examine the therapeutic properties of this MRC. When considering the primary endpoints of this study, treatment efficiency rates were high with an average of 1.5 treatments. This is lower and therefore superior to other MRC studies that report requirements between 2.0 to 3.0 treatments in average (2, 14, 17, 18). Uncritical comparison of these efficiency rates might give the impression that the R-MRC used in this study is superior to other MRCs. However, one must bear in mind that the type of BPPV population being examined is of paramount importance. Most previous MRC studies have included quite complex BPPV populations (CUP subtype-, multicanal-, bilateral-, and treatment retractable BPPV). To a large extent, these interstudy BPPV population characteristic differences might explain the efficiency rate superiority seen with this study. If complete remission of objective findings defined successful treatment, 1.5 treatments were required and if complete subjective remission defined successful treatment, 1.4 treatments were needed in average.

This study also looked at complete resolution rates following one (initial) treatment. In total, 46.7 percent experienced complete resolution of BPPV-related objective findings and subjective symptoms following one treatment. This might seem low in comparison to similar studies that report success rates up to 86.6 percent following one treatment (9). If successful treatment was defined as remission of objective findings alone, another MRC study found a success rate of 65.4% after one treatment of patients with posterior CAN (10). Many studies, however, do not report their criteria defining successful treatment. Our study used quite strict criteria for successful treatment, that may explain these differences. When looking at treatment success rates following one treatment, we found significant differences between the three subgroups of BPPV patients. The Epley maneuver was superior with success rates of 61.7 percent and the 360-degree maneuver was inferior with success rates of 29.4 percent following one treatment. However, these intergroup differences lessened as additional treatments were given over time and became non-significant. One important thing to consider, when doing the inter group comparisons in relation to treatment success rates, is the fact that the vast majority of patients with secondary BPPV [five out of six (83.3%)] was allocated to the group undergoing treatments with the 360-degree somersault maneuver. This might have altered the results, as previous studies have shown the secondary BPPV is more retractable to treatment than primary BPPV (19). When defining treatment success by the strictest criteria (complete remission of both objective findings and subjective symptoms), the 360-degree somersault maneuver behaves poorly on results (significantly worse after one treatment and non-significantly overall). The lack of significance overall might be due to the relatively low number of participants in the three groups, as 74 and 53 percent is a great percentage difference between the Epley- and the 360-degree somersault subgroup (Table 3). An additional explanation for the inferior results with this maneuver might be that up until now no one have defined and described the optimal way of performing the 360-degree somersault maneuver. Must this maneuver be done as one continuous 360-degree turn? What is the optimal total duration of this maneuver (fast or slow turn)? If stops should be included during the maneuver, what is the optimal number of stops during the maneuver and what is the optimal duration of these individual stops?

Evaluation of the amount of subjective relief in total, and with three separate treatment modalities, showed very high success rates. Overall, 99 out of 105 (94.3%) experienced either relief or complete remission of BPPV related symptoms following treatment and there were no significant inter-subgroup differences. These findings are in accordance with previous studies which report subjective relief rates of 91.7% (2), 96.1% (7), and 96.7% (20).

Patient related outcome measures (PROMs) in this study included evaluation of any subjective feeling of vertigo/dizziness by means of fulfillment of the 25-item Dizziness Handicap Inventory (DHI) questionnaire. All post-treatment total- and subcategory scores decreased substantially. Mean differences between overall and subcategory scores pre- and post-treatment were all significant. Overall and subcategory scores, however, did not go down to zero, but total scores were all in the range of the category defined as “no dizziness handicap.” When comparing total mean DHI scores pre- and posttreatment another similar study found total mean DHI scores to drop from 45 to 22 (21).

When considering treatment failures overall, six out of 105 patients (5.7%) experienced no subjective relief or remission of BPPV-related symptoms. Eight out of 105 patients (7.6%) had no relief or remission of objective findings, and two patients (1.9%) had neither subjective nor objective relief or remission following ten treatments. Treatment failures may be defined in different ways. Based upon clinical evaluation, two studies found a 6% (7) and 7.4% (17) overall treatment failure rate if treatment failure was defined as non-successful treatment following a maximum of ten treatments with an MRC. If complete resolution or relief of subjective symptoms was required, an overall treatment failure rate of 3% was reported by West et al. (2) and an overall treatment failure rate of 10% was reported by Li et al. (19). If no relief or remission of objective findings defined treatment failure, a failure rate of 2.5% was found by Tan et al. (13).

### Strengths and weaknesses

This study was carried out as a prospective block randomized study with a thorough description of the methods applied. In general, neither a clear definition of successful BPPV treatment nor consensus on specific prerequisites (PROMs, observations, specific eye monitoring measures etc.) exist. To overcome this matter, we tried to describe the study population in detail and included both objective and subjective outcome measures for easier inter study comparisons.

However, in this study we only evaluated patients directly referred from a GP and exclusively patients diagnosed with posterior CAN or CUP. This means that patients included in this study, supposedly, predominantly presented with uncomplicated BPPV (mono canal, posterior canal, CAN subtype BPPV) that, in general, is very susceptible to treatment. This might have influenced or skewed our results. The fact that our DHI-questionnaire response rates post treatment were not 100 percent might also, theoretically, have altered the results reported with this study.

The R-MRC differs from other MRCs in being portable and by not having predetermined 45-degree specific positioning of the patient in the yaw- and roll axes. Potentially, the R-MRC might therefore prove advantageous for out-of-hospital care (close-to-citizen diagnostics and -treatment) as well as individualized diagnostics and treatment options (based upon patient specific inner ear anatomy). Up until now very few studies have examined the R-MRC. Additional studies are therefore required and should include direct comparisons between diagnostics with traditional BPPV diagnostic tests carried out on an examination table and with the R-MRC, treatment efficiency with traditional treatment maneuvers carried out on an examination table and with the R-MRC, as well as direct comparisons between different MRCs available (diagnostic- as well as therapeutic properties).

## Conclusion

Treatment of patients diagnosed with posterior BPPV with the R-MRC used in this study is very efficient with success rates up to 94.3 percent. The number of treatments required to achieve complete resolution of both objective findings as well as subjective symptoms was 1.5. Three separate BPPV treatment modalities were tested and no significant intergroup success rate differences following up to three treatment attempts were seen. Patient-reported subjective relief in total and with three separate treatment modalities reached 94.3 and between 91.9 to 97.1, respectively. Almost 47 percent of patients experienced complete resolution of both subjective and objective measures following one (first) treatment. All DHI-scores decreased significantly post-treatment. During this study, no adverse- or serious adverse events were registered with the use of this MRC. Therefore, based on the findings in this study, this MRC seems both effective and safe to use.

## Data availability statement

The raw data supporting the conclusions of this article will be made available by the authors, without undue reservation.

## Ethics statement

The studies involving humans were approved by North Denmark Region Committee on Health Research Ethics. The studies were conducted in accordance with the local legislation and institutional requirements. The participants provided their written informed consent to participate in this study. Written informed consent was obtained from the individual(s) for the publication of any potentially identifiable images or data included in this article.

## Author contributions

DH: concept and supervision of project. DH, KD, and MB: design, materials, analysis and/or interpretation, writing manuscript, and critical review. DH, KD, and MB: data collection and/or processing and literature search. All authors contributed to the article and approved the submitted version.

## Conflict of interest

The authors declare that this study was conducted in the absence of any commercial or financial relationships that could be construed as a potential conflict of interest.

## Publisher’s note

All claims expressed in this article are solely those of the authors and do not necessarily represent those of their affiliated organizations, or those of the publisher, the editors and the reviewers. Any product that may be evaluated in this article, or claim that may be made by its manufacturer, is not guaranteed or endorsed by the publisher.
